# Effectiveness of acupuncture for the recovery of gastrointestinal function of patients with gastric cancer in the postoperative period: A protocol for systematic review and meta-analysis

**DOI:** 10.1097/MD.0000000000023950

**Published:** 2021-02-19

**Authors:** Huaiyu Li, Yun Chen, Ziyi Hu, Jiawang Jiang, Renliang Li, Qianjie Qiu, Jing Ye

**Affiliations:** a Jiangxi University of Traditional Chinese Medicine, Nanchang; b First Affiliated Hospital of Gannan Medical University, Ganzhou, China; c The Affiliated Hospital of Jiangxi University of Traditional Chinese Medicine.

**Keywords:** acupuncture, effectiveness, gastric cancer, gastrointestinal function

## Abstract

**Background:**

Gastric cancer (GC) is the most common malignant tumors in the world and surgical resection remains the primary treatment for it. Postoperative patients often suffer from gastrointestinal dysfunction as the most common side effects of surgery for GC patients. Acupuncture has a regulatory effect on gastrointestinal function. We conducted this study to assess the effectiveness of acupuncture on the restoration of gastrointestinal function of postoperative patients with GC.

**Methods:**

Seven electronic databases will be searched from inception to November 2020 to identify any relevant study: Medline, Embase, Cochrane Central Register of Controlled Trials (CENTRAL), China National Knowledge Infrastructure (CNKI), Wanfang Database, Chinese Biomedical Literature Database (CBM), and Chinese Scientific Journal Database (VIP database). No restriction on time and language. The primary outcome measure will be the Time to First Flatus and secondary outcome measures include the time of first defecation and the quality of life (QOL) and the number of patients with abdominal distention. We will use EndNote V.9.1 to screen the eligible literature and the *I*^2^ statistic to assess heterogeneity in the included studies. The meta-analysis will be conducted using the Review Manager (RevMan) software (V.5.3).

**Results:**

Our study aims to systematically assess whether the pooled effects of currently available trials prove effects of acupuncture in improving gastrointestinal function of patients with GC in the postoperative period.

**Conclusion:**

This study will conduct an evaluation about the efficacy of acupuncture for the recovery of gastrointestinal function of patients with GC in the postoperative period, making up for the lack of relevant clinical evidence.

**INPLASY registration number:**

INPLASY2020110066.

## 1 Introduction

Gastric cancer (GC), one of the most common malignant tumors in the world,^[1,2]^ largely derives from the glands of the most superficial layer, or the mucosa, of the stomach.^[3]^ The vast majority of GC are designated as adenocarcinomas from gastric antrum, gastric body and cardia. the pathogenesis of GC is currently multifactorial, and it is mainly related to risk factors such as age, gender, helicobacter pylori, low consumption of fruits and vegetables, and cigarette smoking.^[4]^ According to data from the Global Cancer Epidemiology Database (GLOBOCAN) in 2018, GC, of which the global incidence was 5.7% and the mortality rate was 8.2%, has become the fifth most common cancer in the world and the third leading cause of cancer deaths.^[2]^ Surgical resection currently remains the primary treatment for GC. The early surgical treatment of GC is endoscopic resection, including endoscopic mucosal resection (EMR) and endoscopic submucosal dissection (ESD).^[1]^ A D1 lymphadenectomy is indicated for T1a tumors that not meet the standards for EMR/ESD, and for cT1bN0 tumors that are histologically differentiated type and 1.5 cm or smaller in diameter.^[5]^ The 5-year survival rate of postoperative patients with early GC is proved more than 90.00%.^[6]^ Standard gastrectomy for advanced GC is the main surgical method of radical surgery, which involves removal of at least two-thirds of the stomach and D2 lymph node dissection.^[5]^

Postoperative disturbances of gastrointestinal function are one of the most common side effects of abdominal surgery for cancer.^[7]^ Postoperative patients often experience weakening of gastrointestinal motility, causing progressive accumulation of gastrointestinal secretions. It not only increases the risk of aspiration in patients during the early postoperative period, but exacerbates nutritional deficiencies and even causes systemic Dysfunction.^[8]^ In the past few decades, although some measures related to the promotion of postoperative gastrointestinal function recovery have been used,^[7,9–12]^ the therapy of gastrointestinal function disorders still needs to be continuously improved.

As an indispensable part of Chinese medicine, Acupuncture in recent years has been more accepted by both patients and healthcare providers around the world due to its high safety^[13–15]^ and wide range of uses.^[16]^ Acupuncture has a regulatory effect on gastrointestinal function, widely used in functional constipation FC^[17,18]^, irritable bowel syndrome IBS^[19,20]^, gastroesophageal reflux GER^[21]^and so on. Though many studies have indicated that acupuncture can improve gastrointestinal dysfunction of Patients with GC in the postoperative period, there is a lack of relevant comprehensive assessment about them and so that the overall effect of it on gastrointestinal dysfunction remains unclear. Therefore, it is necessary to conduct this study to make up for the lack of relevant clinical evidence.

## 2 Objectives

The aims are:

1. to evaluate the effectiveness of acupuncture on the recovery of gastrointestinal function of patients with GC in the postoperative period and
2. to provide up-to-date summary of the literature-based references for comprehensively clinical decision-making.

## 3 Methods and analysis

### 3.1 Study registration

The protocol for this review was developed in accordance with the PRISMA-P guidelines and the Cochrane Handbook.^[22,23]^ This protocol has been registered on INPLASY (registration number: INPLASY2020110066: https://inplasy.com/inplasy-2020-11-0066/).

### 3.2 Inclusion criteria

#### 3.2.1 Type of studies

All randomized controlled trials (RCTs) reported will be included without regional and language restrictions. Animal studies, cohort studies, case-controlled studies, case reports and expert experience will be excluded.

#### 3.2.2 Type of participants

All postoperative patients with GC, regardless the age, gender, race, country and GC type.

#### 3.2.3 Type of interventions

All types of acupuncture treatment will be included, such as body acupuncture, electro-acupuncture, auricular acupuncture, warm acupuncture, fire needling, elongated needle and moxibustion. Neither the number of treatments nor the length of treatment will be restricted in this review.

#### 3.2.4 Type of comparators

Control interventions may include one of the following treatment methods: general care, sham acupuncture, placebo, physical/mental training therapy, adjuvant chemotherapy or other pharmacotherapy.

#### 3.2.5 Types of outcome measures

##### 3.2.5.1 Primary outcomes

The primary outcome measure will be the time to first flatus.

##### 3.2.5.2 Secondary outcomes

Secondary outcome measures include the time of first defecation and the quality of life (QOL). The number of patients with abdominal distention will be employed as one of adverse events (AEs).

### 3.3 Exclusion criteria

- RCTs comparing 2 different types of acupuncture;
- Non-randomized controlled trials
- Duplicated data
- Invalid outcome indexes.

### 3.4 Search methods for identification of studies

#### 3.4.1 Electronic searches

The following electronic databases will be searched from inception to November 31, 2020 to identify any relevant study: Medline, Embase, Cochrane Central Register of Controlled Trials (CENTRAL), China National Knowledge Infrastructure (CNKI), Wanfang Database, Chinese Biomedical Literature Database (CBM), and Chinese Scientific Journal Database (VIP database). The key words include “acupuncture”, “body acupuncture”, “electro-acupuncture”, “warm acupuncture”, “auricular acupuncture”, “fire needling”, “elongated needle” and “gastric cancer”. An equivalent translation of the same search terms will be used to search in the Chinese databases. No restriction on time and language will be applied. The search strategy of PubMed is shown in Table 1.

**Table 1:** Search strategy used in PubMed database.

Order	Search items
#1	((((((((((((((((((Stomach Neoplasms) OR (Stomach Neoplasms)) OR (Stomach Neoplasm)) OR (Neoplasms, Stomach)) OR (Gastric Neoplasms)) OR (Gastric Neoplasm)) OR (Neoplasm, Gastric)) OR (Neoplasms, Gastric)) OR (Cancer of Stomach)) OR (Stomach Cancers)) OR (Gastric Cancer)) OR (Cancer, Gastric)) OR (Cancers, Gastric)) OR (Gastric Cancers)) OR (Stomach Cancer)) OR (Cancer, Stomach)) OR (Cancers, Stomach)) OR (Cancer of the Stomach)) OR (Gastric Cancer, Familial Diffuse) [All Fields]
#2	((((((((((((((((Acupuncture) OR (acupuncture therapy)) OR (Electroacupuncture)) OR (electroacupuncture therapy)) OR (manual acupuncture)) OR (moxibustion)) OR (Acupuncture, Ear)) OR (Acupunctures, Ear)) OR (Ear Acupunctures)) OR (Auricular Acupuncture)) OR (Ear Acupuncture)) OR (Acupuncture, Auricular)) OR (Acupunctures, Auricular)) OR (Auricular Acupunctures)) OR (warm acupuncture)) OR (fire needling)) OR (elongated needle) [All Fields]
#3	(((((Acupuncture Points) OR (Acupuncture Point)) OR (Point, Acupuncture)) OR (Points, Acupuncture)) OR (Acupoints)) OR (Acupoint) [All Fields]
#4	randomized controlled trial[Publication Type] OR randomized [Title/Abstract] OR placebo [Title/Abstract]
#5	#1 AND #2 AND #3 AND #4

#### 3.4.2 Searching other resources

We will search The WHO International Clinical Trials Registry Platform (ICTRP), Chinese Clinical Trial Registry, Clinical Trials gov to identify any potentially eligible studies.

### 3.5 Selection of studies

**Figure 1:**
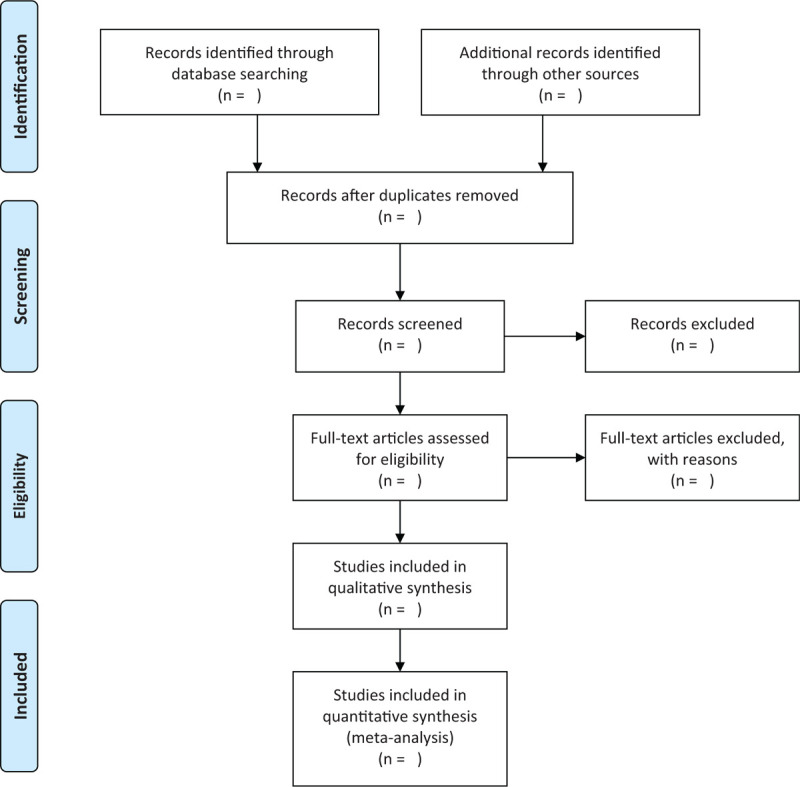
Flowchart of literature selection.

We will use EndNote V.9.1 to screen the eligible literature through electronic/manual-based steps. The process of selecting the search results will be independently performed by 2 reviewers according to the inclusion criteria. They will review and screen the titles and abstracts retrieved by literature search to exclude irrelevant trials. The causes of both selections will be documented and full texts will be obtained and checked for further evaluation if necessary. Any disagreement between reviewers will be resolved via discussion and consensus with a third reviewer of the review team. The selection process will be showed in a PRISMA flow diagram (Fig. 1).

### 3.6 Data extraction and management

All data extraction will be carried out independently by 2 reviewers using a pre-designed extraction form. Information extracted will include first author, publication year, study design, characteristics of participants, type of treatments, outcome measures, and adverse events. Disagreements between reviewers in the process of data extraction were resolved by discussing with a third reviewer. Incomplete data will be provided by contacting corresponding authors.

### 3.7 Assessment of the methodological quality

The Risk of bias assessment form developed by the Cochrane Collaboration will be used as the assessment of methodological quality by 2 reviewers.^[23]^ It includes the following 7 domains: random sequence generation, allocation concealment, blinding of participants and personnel, blinding of outcome assessment, incomplete outcome data, selective reporting and other sources of bias. Each domain includes a judgment of low (meeting all criteria), high (meeting none of criteria) and unclear (insufficient information to judge) risk of bias according to information provided by authors. Disagreements between reviewers will be resolved through discussion with a third reviewer.

### 3.8 Measures of treatment effect

Mean difference (MD) or standardized mean difference (SMD) will be used to evaluate the treatment effect for continuous outcomes and the risk ratio (RR) will be used to assess the treatment effect for dichotomous outcomes. Ninety five percentages of the confidence intervals (CIs) will be used as an effective size for the analysis.

### 3.9 Dealing with missing data

For studies having insufficient data to enter in the meta-analysis, we will attempt to contact authors of the study to obtain missing data. If we cannot contact the original authors, the studies will be excluded from the data synthesis.

### 3.10 Assessment of heterogeneity

The *I*^2^ statistic will be calculated to check the possibility of Statistical heterogeneity in the outcomes of the included studies. The results of the *I*^2^ statistic, which determine the using of fixed-effects model or random-effects model, cover unimportant heterogeneity (0%–40%), moderate heterogeneity (30%–60%), substantial heterogeneity (50%–90%) and considerable heterogeneity (75%–100%). If the conclusion of high levels of heterogeneity in the included studies is drawn, we will seek to account for the source of heterogeneity by sensitivity analysis or subgroup analysis.

### 3.11 Data synthesis

The meta-analysis will be conducted using the Review Manager (RevMan) software (V.5.3). If the result of heterogeneity in *I*^2^ < 40%, the fixed-effects model will be used for data synthesis and analysis; If *I*^2^ ≥ 40% and <75%, the random-effects model will be implied; If *I*^2^ ≥ 75%, it means there is considerable heterogeneity between studies and the narrative summary of the studies will be done.

### 3.12 Subgroup analysis

If there is substantial heterogeneity between the study results, subgroup analysis will be performed, following items will be considered: type of acupuncture, gender, age, and outcome styles.

### 3.13 Sensitivity analysis

When sufficient data are available, sensitivity analysis will be conducted to verify the robustness of the results. It includes the impact of methodological quality, study design and sample size.

### 3.14 Grading the quality of evidence

Two reviewers will independently use the Grading of Recommendations Assessment, Development and Evaluation (GRADE), which evaluates the quality of evidence as “high”, “moderate”, “low”, or “very low”, to assess the quality of evidence.^[24]^

### 3.15 Ethics and dissemination

The study will be published in a peer-reviewed journal or relevant conference. No ethical approval is required. The results of the study will provide potential guidance in advancing the therapeutic strategy of patients with GC in the postoperative period.

## 4 Discussion

Although surgical treatment is the primary choice for GC patients, the majority of them often suffer from gastrointestinal dysfunction in the postoperative period. It has been suggested that postoperative gastrointestinal dysfunction will cause delayed passage of gas and stool, postoperative pain and resumption of oral feeding, resulting in prolonged postoperative hospital stay and increased medical costs.^[25]^ Delayed passage of gas and stool is the most common postoperative complications among them. Acupuncture, one of the major treatment modalities in Chinese medicine, is increasingly used in the world. Several RCT studies recently have provided relevant evidence to estimate the effectiveness of acupuncture for treating patients with GC in the postoperative period. Therefore, our current study aims to systematically assess whether the pooled effects of currently available trials prove effects of acupuncture in improving gastrointestinal function of patients with GC in the postoperative period.

## Author contributions

**Conceptualization:** Huaiyu Li, Jing Ye.

**Data curation:** Huaiyu Li, Ziyi Hu, Jiawang Jiang.

**Formal analysis:** Yun Chen, Renliang Li, Qianjie Qiu.

**Methodology:** Yun Chen, Ziyi Hu, Jing Ye.

**Software:** Jiawang Jiang, Renliang Li, Qianjie Qiu, Jing Ye.

**Supervision:** Jiawang Jiang.

**Writing – original draft:** Huaiyu Li, Jiawang Jiang, Jing Ye.

**Writing – review & editing:** Renliang Li, Qianjie Qiu, Jing Ye.
